# Successful Resection of Transverse Colon Cancer Invading a Reconstructed Jejunum After Pancreaticoduodenectomy: A Case Report

**DOI:** 10.1002/ccr3.73884

**Published:** 2026-11-03

**Authors:** Kohei Oyamada, Masaya Hiyoshi, Kotaro Nishida, Takayuki Okuno, Hiroshi Kawasaki, Yusuke Kyoden

**Affiliations:** ^1^ Department of Gastrointestinal Surgery Ibaraki Cancer Center, Ibaraki Prefectural Central Hospital Kasama Ibaraki Japan; ^2^ Department of Gastrointestinal Surgery Japanese Red Cross Omori Hospital Ota‐ku Tokyo Japan

**Keywords:** En bloc resection, hepatectomy, invasion, jejunum, neoadjuvant chemotherapy, pancreaticoduodenectomy, transverse colon cancer

## Abstract

The treatment of locally advanced colon cancer is surgically challenging, particularly in patients with prior pancreaticoduodenectomy. Careful assessment after neoadjuvant chemotherapy may enable curative resection while preserving pancreaticobiliary reconstruction. As the postoperative prognosis for pancreaticoduodenectomy improves, similar cases can be expected to increase.

**Abbreviations**
CapeOX+BV: capecitabine and oxaliplatin plus bevacizumab regimen
CT: computed tomography
NAC: neoadjuvant chemotherapy
PD: pancreaticoduodenectomy
PET: positron emission tomography
PPPD: pylorus‐preserving pancreaticoduodenectomy
UICC: Union for International Cancer Control

## 1 Introduction

Locally advanced colon cancer frequently requires multivisceral resection [1]. Its management becomes more complicated in patients who have previously undergone pancreaticoduodenectomy (PD) due to postoperative adhesions, altered anatomy [2] and the potential need for redo pancreaticobiliary reconstruction. Surgical resection is the only curative option, but the optimal surgical strategy for colon cancer invading a reconstructed jejunal limb after PD remains unclear. We report a case of transverse colon cancer that invaded both the liver and the elevated jejunal loops reconstructed during pylorus‐preserving PD (PPPD). This case demonstrates the importance of careful surgical assessment after neoadjuvant chemotherapy (NAC) to achieve curative resection while avoiding unnecessary redo pancreaticobiliary reconstruction.

## 2 Case Presentation

A 77‐year‐old woman was referred to our hospital with a 45‐mm mass detected by abdominal ultrasound at the previous clinic. She had undergone PPPD 20 years earlier for an intraductal papillary mucinous neoplasm. Contrast‐enhanced computed tomography (CT) identified a tumor of the transverse colon invading the lateral hepatic segment and elevated jejunum at multiple sites (Figure 1). Positron emission tomography (PET)/CT revealed no obvious distant metastasis. A colonoscopy identified a circumferential Type 2 lesion in the transverse colon, which almost occluded the lumen (Figure 2). A biopsy from the tumor revealed well‐differentiated adenocarcinoma. Immunohistochemical analysis of mismatch repair proteins and microsatellite instability testing showed no abnormalities. Initial blood tests showed a carcinoembryonic antigen (CEA) level of 9.8 ng/mL and a cancer antigen 19–9 (CA19‐9) level of 33.1 U/mL. Based on the 8th edition of the Union for International Cancer Control (UICC) guidelines, we diagnosed clinical stage IIIC T4bN2bM0 transverse colon cancer invading the liver and the elevated jejunum.

**FIGURE 1:**
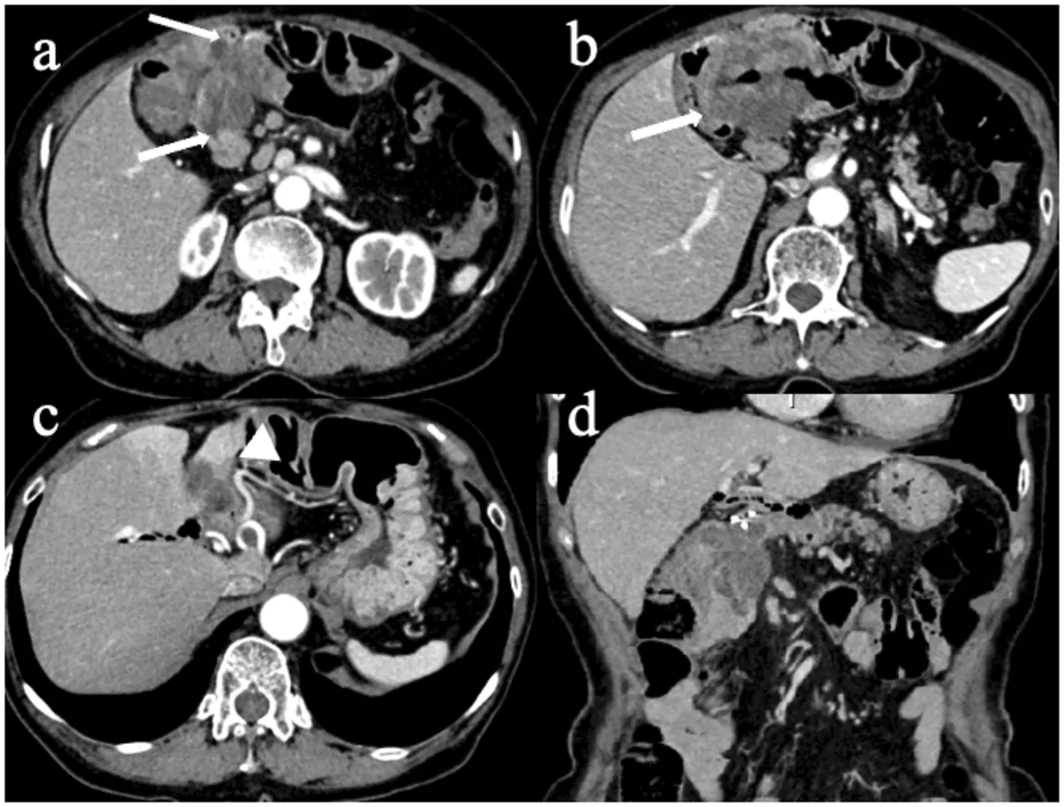
Contrast‐enhanced CT scan findings from our patient at initial examination. The image shows a large tumor of the transverse colon. The tumor invades the elevated jejunum at multiple sites (arrow) (a, b, d) and the lateral hepatic segment (triangle) (c). CT, computed tomography.

**FIGURE 2:**
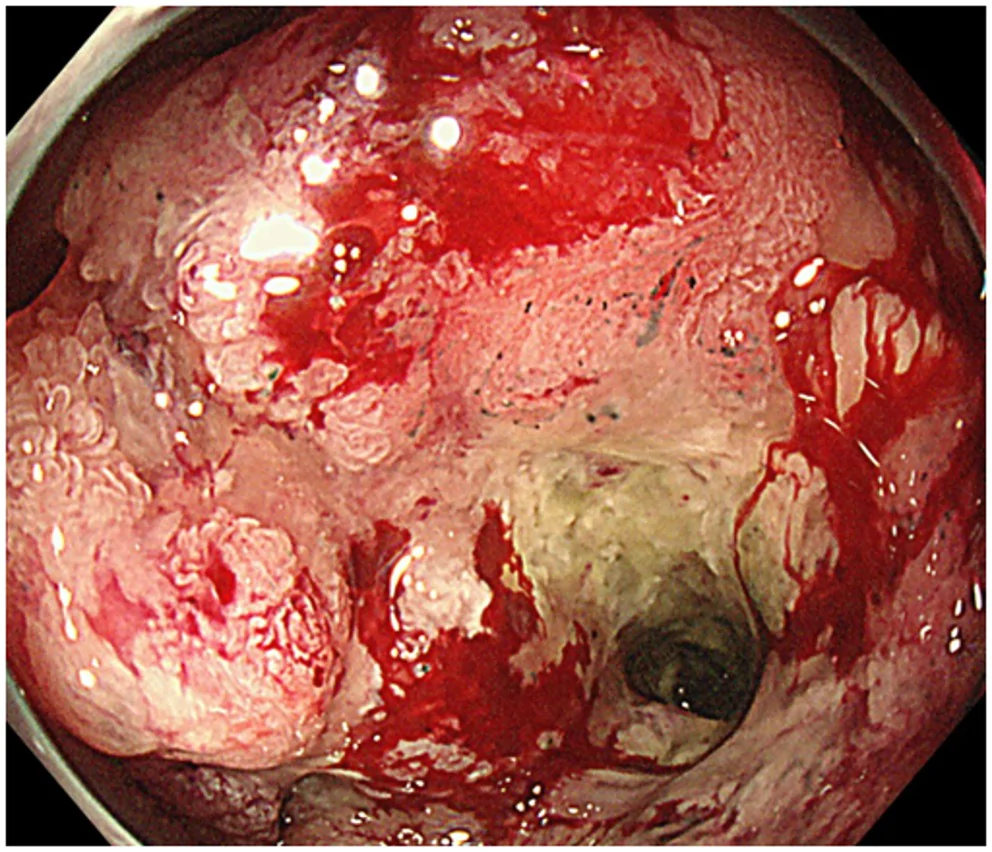
Lower gastrointestinal endoscopy findings from our patient at initial examination. A colonoscopy revealed a circumferential tumor of the transverse colon.

We decided to proceed with ileostomy and NAC because upfront surgery would require extensive multivisceral organ resection. After three courses of the capecitabine and oxaliplatin with bevacizumab (CapeOX+BV) regimen, a PET/CT scan confirmed a decrease in tumor size and diminished fludeoxyglucose uptake (Figure 3). The patient's serum CEA level was reduced to 4.2 ng/mL. We felt it was now appropriate to perform surgery. During surgery, the transverse colon cancer was found to invade the liver and the elevated jejunum (Figure 4). However, the elevated jejunum vessels, including those created by pancreatojejunostomy and choledochojejunostomy, were unaffected. We performed three limited jejunal resections and anastomoses at the affected sites (Figures 5 and 6). This was followed by right hemicolectomy and limited resection of the liver and elevated jejunum. Postoperative histological findings showed that the cancer had invaded the elevated jejunum, but no liver invasion was apparent microscopically. None of the 23 retrieved lymph nodes showed signs of metastasis, and the surgical margins were clear of tumor cells. We finally diagnosed posttherapy pathological stage IIC T4bN0M0 per 8th edition UICC guidelines.

**FIGURE 3:**
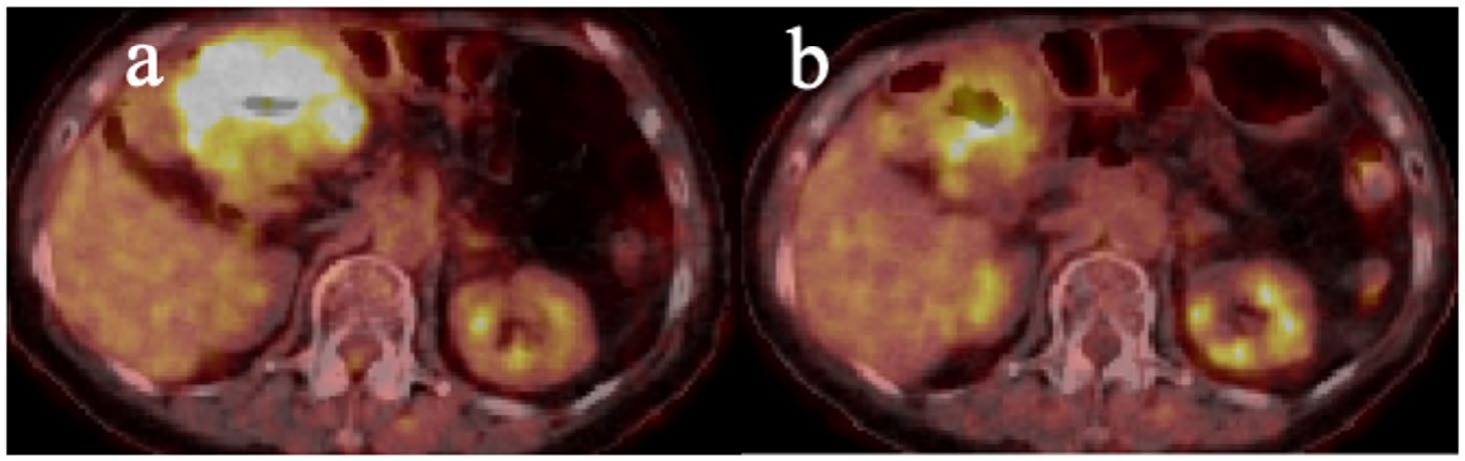
PET/CT scan findings from our patient. (a) Scan at initial examination; (b) Scan after three courses of CapeOX+BV therapy. Comparison of the PET/CT scans before and after three courses of CapeOX+BV neoadjuvant chemotherapy confirmed that the tumor had shrunk. There was also diminished FDG uptake. CapeOX+BV, capecitabine and oxaliplatin plus bevacizumab regimen; CT, computed tomography; FDG, fludeoxyglucose; PET, positron emission tomography.

**FIGURE 4:**
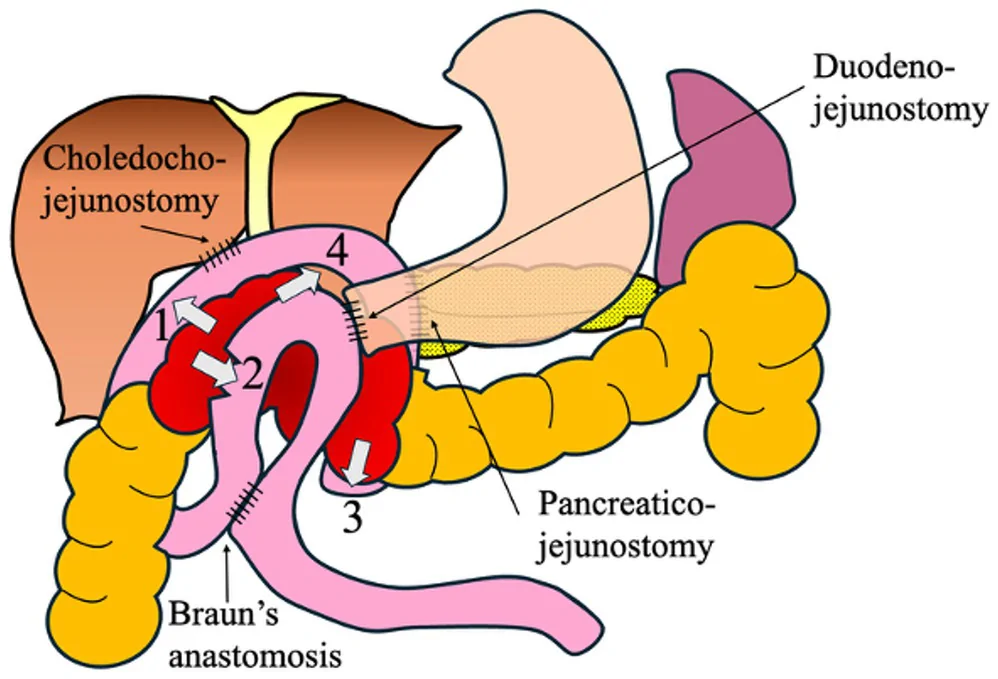
Illustration of intraoperative view/findings. Tumor invasion was observed in the elevated jejunal limb at multiple locations, including the segment between the choledochojejunostomy and the Braun's anastomosis (←1), the segment between the Braun's anastomosis and the duodenojejunostomy (←2), and the blind end (←3). The tumor also invaded the liver (←4). The jejunal invasion had not reached any of the anastomoses, and the biliary/pancreatic reconstructions were preserved.

**FIGURE 5:**
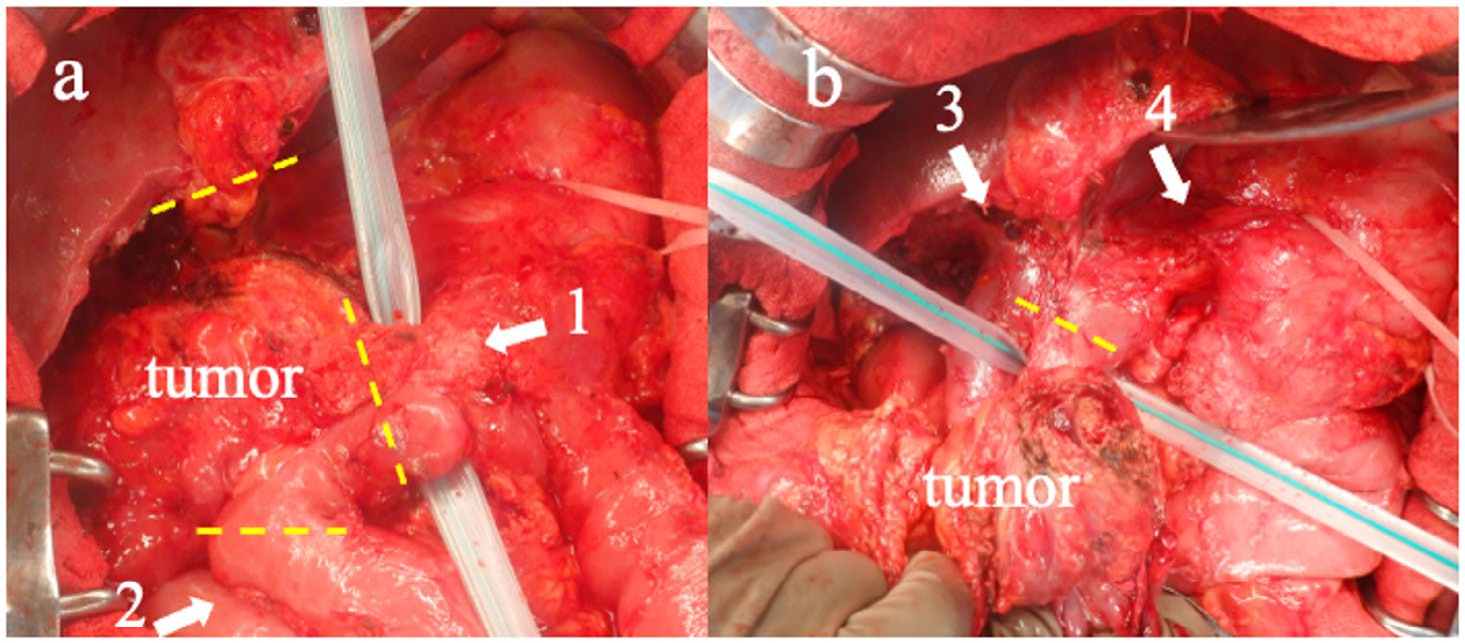
Intraoperative photographs of transverse colon cancer resection in our patient. (a) We found that duodenojejunostomy (←1) and Braun's anastomosis (←2) were safe after a partial hepatectomy; (b) the previously performed choledochojejunostomy (←3) and pancreatojejunostomy (←4) were preserved. The yellow lines demarcate the dissection performed.

**FIGURE 6:**
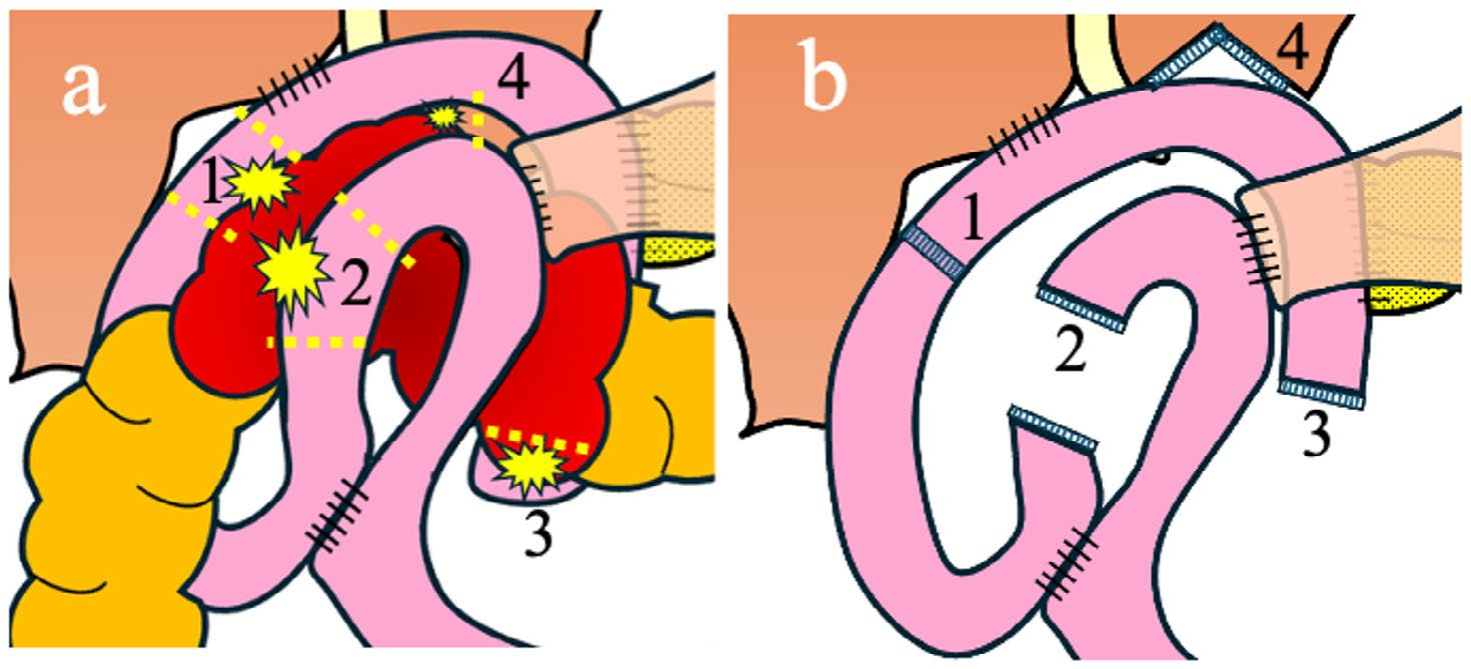
Illustration of the intraoperative view/findings from our patient. (a) Tumor invasion was observed at multiple locations in the elevated jejunal limb; (b) The patient underwent right hemicolectomy, partial hepatectomy, partial jejunal resection, and ileostomy closure. Partial jejunal resection and anastomoses were performed between the choledochojejunostomy and Braun's anastomosis (←1), but no reconstruction was required as food passage was preserved (←2) (←3). Partial hepatectomy was performed (←4). The yellow lines show the dissection performed in the partial hepatectomy and the partial jejunal resections.

The patient's postoperative course was uneventful, and she was discharged on postoperative day 11. She underwent a further four courses of CapeOX+BV as adjuvant chemotherapy. At the 2‐year follow‐up, there had been no recurrence.

## 3 Discussion

Colorectal cancer is one of the most common malignancies worldwide, with epidemiological data indicating an increasing incidence, particularly in younger individuals [3]. When colorectal cancer is suspected to have invaded adjacent organs, especially previously reconstructed organs, combining organ preservation with complete curative resection is the primary objective. However, upfront surgery is technically challenging or contraindicated [1, 2]. Currently, there are no established guidelines or standard treatments for locally advanced cases [4].

At present, NAC is among the most feasible options for locally advanced colon cancer [5]. It is known to contribute to pathological downstaging and to reduce the risk of recurrence [6]. Recent research has reported significant reductions in tumor volume, averaging 62% [7]. NAC also minimizes the extent of invasion and provides sufficient tumor margins [8]. Furthermore, NAC provides significantly improved rates of 5‐year overall survival (76.3% vs. 69.2%) and disease‐free survival (67.2% vs. 60.1%) compared with upfront surgery [9]. NAC has been shown to improve the resection rate even in initially unresectable cases with multiple distant metastases [10]. Furthermore, it has been shown to reduce the extent of resection required at sites such as the abdominal wall [8], the duodenum, and the kidneys [11]. Thus, NAC is a safe and feasible option in the management of locally advanced colon cancer.

In this case, NAC effectively decreased tumor size and tumor marker levels. The tumor was initially suspected to have invaded the reconstructed jejunal limb and its vessels, as well as other organs. Theoretically, curative resection at that point would have required complete resection of the elevated jejunum as well as reanastomosis of pancreatojejunostomy and choledochojejunostomy. Such procedures are highly invasive and may substantially increase postoperative morbidity. To avoid excessive surgery, we decided to precede surgery with NAC. Consequently, we were able to utilize acceptable procedures such as limited resection of the liver and the elevated jejunal limb without redo pancreaticobiliary reconstruction. The absence of direct involvement of the pancreatojejunostomy and choledochojejunostomy allowed preservation of pancreaticobiliary reconstruction. It is difficult to determine the extent to which the NAC contributed to reducing the resection required. The optimum duration of NAC prior to surgery is also unknown, and there are no established findings indicative of the point at which surgery should be performed. Nevertheless, NAC contributed to the curative success of the resection in this case, improving the patient's postoperative prognosis. Avoiding unnecessary redo pancreaticobiliary reconstruction while maintaining curative intent should be a key consideration when treating colon cancer invading a reconstructed jejunal limb after PD.

The resection rate for borderline pancreatic cancer has doubled from 8.3% to 16.6% over the last two decades [12]. Moreover, the prognosis of patients with pancreatic cancer and distal bile duct cancer has improved due to treatment advances [13, 14]. As long‐term survival after PD continues to improve, surgeons are increasingly likely to encounter secondary malignancies arising in patients with altered postoperative anatomy. This case highlights the importance of evaluating reconstructed anatomy and considering organ‐preserving strategies. We hope this report will aid the treatment choices of clinicians with similar cases.

## 4 Conclusion

As the prognosis for PD improves, cases of secondary colorectal cancer can be expected to increase. This case highlights the importance of careful assessment of reconstructed anatomy after NAC to achieve curative resection while preserving pancreaticobiliary reconstruction and avoiding unnecessary redo reconstruction.

## Author Contributions

**Kohei Oyamada:** conceptualization, data curation, resources, software, visualization, writing – original draft. **Masaya Hiyoshi:** resources, visualization, writing – review and editing. **Kotaro Nishida:** investigation. **Takayuki Okuno:** project administration, writing – review and editing. **Hiroshi Kawasaki:** methodology, resources. **Yusuke Kyoden:** supervision, writing – review and editing.

## Funding

The authors have nothing to report.

## Ethics Statement

As a single‐case report with the patient's signed consent, no other ethical review was required.

## Consent

Written informed consent was obtained from the patient for the publication of this case report.

## Conflicts of Interest

The authors declare no conflicts of interest.

## Data Availability

The authors declare that all data within this article are available upon reasonable request. The data supporting the findings of this study are available from the corresponding author upon reasonable request.
